# Parental educational attainment as an indicator of socioeconomic status and risk of childhood cancers

**DOI:** 10.1038/sj.bjc.6605732

**Published:** 2010-06-08

**Authors:** S E Carozza, S E Puumala, E J Chow, E E Fox, S Horel, K J Johnson, C C McLaughlin, P Reynolds, J Von Behren, B A Mueller, L G Spector

**Affiliations:** 1 Department of Public Health, College of Health and Human Sciences, Oregon State University, Corvallis, OR 97333, USA; 2 Department of Pediatrics, Division of Epidemiology/Clinical Research, University of Minnesota, Minneapolis, MN 55455, USA; 3 Department of Public Health Sciences, Fred Hutchinson Cancer Research Center, 1100 Fairview Avenue N, M4-C308, PO Box 19024, Seattle WA 98109, USA; 4 Center for Clinical and Translational Sciences, University of Texas Health Science Center, Houston, TX 77030, USA; 5 School of Rural Public Health, Texas A&M Health Sciences Center, College Station, TX 78740, USA; 6 New York State Cancer Registry, New York State Department of Health, Corning Tower Room 536, Empire State Plaza, Albany, NY 12237-0679, USA; 7 Northern California Cancer Center, 2201 Walnut Avenue, Suite 300, Fremont, CA 94538, USA; 8 Masonic Cancer Center, University of Minnesota, Mayo Mail Code 806, 420 Delaware Street SE, Minneapolis, MN 55455, USA

**Keywords:** childhood cancer, socioeconomic status, epidemiology

## Abstract

**Background:**

Little has been reported on socioeconomic (SES) patterns of risk for most forms of childhood cancer.

**Methods:**

Population-based case–control data from epidemiological studies of childhood cancer conducted in five US states were pooled and associations of maternal, paternal and household educational attainment with childhood cancers were analysed. Odds ratios (ORs) and 95% confidence intervals were estimated using logistic regression, controlling for confounders.

**Results:**

Although there was no association with parental education for the majority of cancers evaluated, there was an indication of a positive association with lower education for Hodgkin's and Burkitt's lymphoma and Wilm's tumour, with the ORs ranging from 1.5 to >3.0 times that of more educated parents. A possible protective effect was seen for lower parental education and astrocytoma and hepatoblastoma, with ORs reduced by 30 to 40%.

**Conclusions:**

These study results should be viewed as exploratory because of the broad nature of the SES assessment, but they give some indication that childhood cancer studies might benefit from a more thorough assessment of SES.

There is ample evidence that poorer socioeconomic circumstances are linked to poorer health (Seeman and Crimmins, 2001; Galobardes *et al*, 2007). Socioeconomic status (SES) is complex, incorporating aspects of both availability of resources (education, income and wealth) and standing in the hierarchy of a society. SES measures may indicate a variety of health-related factors, including occupational exposures, dietary patterns, residential environmental exposures, exposures to infectious agents, and the knowledge of and adherence to healthy lifestyles (Krieger *et al*, 1997; Braveman *et al*, 2005; Galobardes *et al*, 2007).

A possible role of parental SES in childhood cancers has been investigated for childhood leukaemias, but without conclusive results, with associations varying by study design, time period, SES measure used and whether this was at the individual or family level *vs* neighbourhood or higher grouping (Poole *et al*, 2006; Adam *et al*, 2008). There is little published data on SES patterns of risk for other forms of childhood cancer, most studies tending to consider SES data, typically parental education, only to control for potential confounding. Using a large pooled data set of childhood cancers from five US states, we conducted an exploratory analysis of the effect of SES, as estimated by parental educational attainment, on incidence of specific childhood cancers.

## Material and methods

Details of the study population have been published elsewhere (Puumala *et al*, 2009). Briefly, population-based case–control data from studies conducted in California, Minnesota, New York (excluding New York City), Texas and Washington states (Reynolds *et al*, 2002; McLaughlin *et al*, 2006; Podvin *et al*, 2006; Puumala *et al*, 2008; Carozza *et al*, 2009) were pooled, with birth dates of subjects ranging from 1970 to 2004. For each study, cases of childhood cancer identified in the population-based state cancer registry were matched to state vital records to capture birth certificate data (Jaro, 1995). Cases were classified according to the International Classification of Childhood Cancer third edition (Steliarova-Foucher *et al*, 2005). Controls (frequency matched on delivery year in all states except California, where data were individually matched) were randomly selected from the state birth records, with case/control ratios ranging from 1 : 1 to 1 : 10. The final pooled data set consisted of 17 672 cases and 57 966 controls. Human Subjects Protection Committee approvals were obtained from the institutional review boards of all the participating institutions including each state's health department.

Parental educational data were not available for all years in three states (California, Texas and Washington). Study subjects born in years for which there was no collection of parental educational data were excluded from the pooled data set, resulting in a total of 12 665 cases and 39 472 controls available for analysis. New York state collected education as a categorical variable between 1988 and 1990, which resulted in 643 cases and 1438 controls with maternal education, and 619 cases and 1261 controls with paternal education in categories only. Maternal and paternal education was assigned according to the highest completed level of education. Household education level was determined as either the highest parental education level attained or the highest education level of one parent if the other parent's information was not listed. Educational attainment was analysed by categories typically associated with credentials: <12 years (did not complete high school degree); 12 years (completed high school degree); 13–16 years (Associates or Bachelor degree equivalents); 17 years or more (graduate or professional training). Analyses were also performed to evaluate associations for children whose birth certificate included only maternal data (i.e. missing all paternal data), which potentially indicated a household of low SES at the time of birth (Tan *et al*, 2004). Child-level characteristics retrieved from birth certificate data, included in the evaluation of potential confounding, were birth weight, gestational age, plurality, sex, birth order and year of birth. Parent-level characteristics included maternal and paternal age and race/ethnicity.

### Statistical analysis

Analyses were performed using SAS 9.1. (SAS Institute, Cary, NC, USA). Odds ratios (ORs) and two-sided 95% confidence intervals (CIs) were obtained using unconditional logistic regression, with individual matching in the California data set broken to allow the use of this method. All models included birth weight, gestational age, plurality, sex, birth order, year of birth, maternal age and maternal race/ethnicity as well as state of birth. ORs for educational attainment of mother, father and household are presented for all cancers combined and for International Classification of Childhood Cancer third edition cancer sites with >200 cases in the data set (excluding heterogeneous ‘other’ and unspecified tumour categories). As higher SES is generally beneficial when considering health outcomes, educational attainment of 17 years or more served as the referent category in the models. To assess the association between the different types of educational measurement (maternal, paternal and household), Pearson's correlation coefficient was calculated for each pair. Correlation was calculated overall and within maternal race/ethnicity groups. Additionally, ORs and 95% CIs were calculated for children with only maternal information recorded on the birth certificate compared with children having data for both parents.

To evaluate whether associations varied according to age at diagnosis, age-specific ORs and 95% CIs also were calculated for cancer types with sufficient number of study subjects. Categories of 0–4 and 5–14 years of age were created for most cancers. A category for infants (i.e. ages <1 year) was also created for leukaemias. Because of a lack of older cases, categories of <2 years and 2 years or greater were used for sympathetic nervous system tumours, retinoblastomas, renal tumours, hepatic tumours and soft tissue sarcomas.

## Results

Cases and controls varied slightly in maternal and paternal race/ethnicity, with more cases than controls having Hispanic parents (Table 1). Race/ethnicity reporting was also more complete for cases than for controls. The average age at diagnosis was 4.5 years and slightly more than half of both cases and controls were males. Almost all the children were singletons and 41% were the first live birth for the mother. The majority of parents were non-Hispanic white. Approximately one-third of the mothers were less than 25 years of age at the time of child's birth, with fathers being slightly older.

When considering all study subjects together, the three measures of educational attainment were strongly correlated, however, this masked substantial differences by race/ethnicity (Table 2). Correlation between maternal and paternal education was lowest among non-Hispanic black parents (*r*=0.45).

For all cancers and for most specific types, no association was seen with the three educational measures (Table 3; Supplementary Table 3). A two-fold increased OR was seen for Hodgkin's lymphoma for maternal education <12 years (OR=2.03 (95% CI: 0.87, 4.77)) and for household <12 years (OR=2.17 (95% CI: 1.07, 4.38)), and a three-fold increased OR was seen for Burkitt's lymphoma for maternal education <12 years (OR=3.32 (95% CI: 1.26, 8.71)). In contrast, the association was significantly reduced for the same category for astrocytoma (OR_maternal <12 years_=0.70 (95% CI: 0.51, 0.95)). A significantly decreased OR (OR=0.59) for hepatoblastoma was also seen for maternal education of 12 years and 13–16 years.

A significantly increased OR was seen for paternal high school educational attainment and retinoblastoma (OR=1.45 (95% CI: 1.01, 2.10)), but there was no consistently increased pattern overall. A significant OR of 1.32 was seen for total renal tumours and both paternal and household high school education level. Within renal tumours, Wilm's tumour incidence was significantly increased for paternal education less than high school (OR=1.45 (95% CI: 1.04, 2.02)) and high school education (OR=1.39 (95% CI: 1.06, 1.83)), as well as for the household high school category (OR=1.36 (95% CI: 1.05, 1.75)). Almost two-fold (but non-significant) increase in ORs was seen for the lowest educational attainment category and Ewing's sarcoma (OR_paternal_=1.86 (95% CI: 0.73, 4.72)) and gonadal germ cell tumours (OR_maternal_=1.99 (95% CI: 0.87, 4.56)).

A total of 920 of the cases (7.3%) and 3168 of the controls (8.0%) were missing all paternal data but comparing their ORs with data on the child's father as the SES measure, most sites showed no association (Table 4). A significantly increased OR was seen for rhabdomyosarcoma (OR=1.59; 95% CI (1.11, 2.29)), and an appreciably (but not significantly) decreased OR was seen for Burkitt's lymphoma (OR=0.47; 95% CI (0.20, 1.12)). No consistent statistically significant pattern was seen when evaluating point estimates for younger and older children separately, and the ORs generally reflected those seen for the all-ages data (data not shown).

## Discussion

For the majority of childhood cancers evaluated, no association, either increasing or decreasing, was observed with the level of parental educational attainment. There was an indication of a positive association with lower educational attainment for both Hodgkin's and Burkitt's lymphomas and for Wilm's tumour, with ORs of 1.5 to greater than 3.0 times that of more educated parents. In contrast, a protective effect of lower parental education was suggested for both astrocytoma and hepatoblastoma, with ORs being reduced by 30% to 40% compared with those whose parents were more educated. A significantly increased association was seen for rhabdomyosarcoma only when comparing children whose birth certificates had no paternal data with those that did.

The main strength of this study is the large sample size allowing the assessment of SES in many specific histological types; it therefore adds appreciably to the information on rare childhood cancers. Additional strengths include our use of population-based cancer surveillance data, of prospectively collected birth records and use of population-based passive control selection.

Several limitations should be noted, including the effects of multiple comparisons; significant results in the subgroup analyses in particular could be because of chance alone. There is also the potential for error in birth certificate-based data, as items on these records have varying levels of accuracy. In a study comparing mothers’ responses in a national natality survey with those on linked birth certificates, a 77% concordance was reported on maternal education overall between the two data sources, with an additional 15% reporting a difference of only 1 year (Querec, 1980). A 72% concordance was found for reported paternal education. Higher agreement was found when considering the education data in categories, with 90% agreement for 12 years (i.e. high school diploma) of maternal education and 86% for the same cut-point in paternal education. The report concluded that grouping educational data into categories marked by certificates or diplomas was likely to result in the least bias. Most variables used to adjust for confounding, such as maternal age, birth plurality and birth weight, have consistently been found to be accurate in validation studies (Northam and Knapp, 2006).

Interpretation of our results must also be viewed within the context of limited but pertinent information about SES conveyed by parental educational level. Numerous studies have confirmed that the health and welfare of children is associated with parental educational attainment, with parental education level often a stronger predictor of children's health than family income, number of parents or size of the family (Zill, 1996). With respect to any SES-related risk factors operating in childhood cancers and whether they are captured by educational measures, it seems reasonable to hypothesize that education can influence the likelihood of particular occupational exposures of either parent, for example, through the employment opportunities available to those with and without high school diplomas. Specific health behaviours are influenced by educational level as well, such as awareness of and access to prenatal care, smoking tobacco products, specific dietary choices and compliance with immunization schedules (Zill, 1996). All these factors may be associated with risk for specific types of childhood cancers, with components of one or more acting either as initiators and/or promoters of carcinogenesis.

There is no single gold standard indicator of SES (Krieger *et al*, 1997; Daly *et al*, 2002; Braveman *et al*, 2005; Galobardes *et al*, 2006) and education is a commonly used indicator of overall SES in epidemiology; it is an attempt to capture knowledge-related assets (Galobardes *et al*, 2006). Household educational attainment has been recommended for studies of child development (Hauser, 1994) and may be the most appropriate approach for childhood diseases. A simple determination of years of schooling completed, however, contains no information about its quality, which may be important for health outcomes specifically related to knowledge, cognitive skills and analytical abilities. Quality of education may be less important, however, as a broad indicator of SES (Galobardes *et al*, 2006), as we propose in this study. Finally, our analysis considers SES around the time of birth, but for some cancers, SES during childhood or nearer to the time of diagnosis might be more relevant.

Our findings are consistent with a long-recognized association between increased Hodgkin's lymphoma incidence in childhood and markers of lower socioeconomic class (Correa and O’Conor, 1971; Gutensohn and Shapiro, 1982). In contrast, Hodgkin's lymphoma in young adults has been associated with markers of higher early childhood SES, which has been thought to reflect responses to infection, with the more protected environment associated with higher SES resulting in a delayed exposure and response to the infectious agent (Stiller, 1998). Recent research has implicated the Epstein–Barr virus (EBV) as a possible infectious agent for Hodgkin's lymphoma in children (Kutok and Wang, 2006). EBV seroconversion tends to occur much earlier in low SES populations, in which crowded housing predominates (Henle and Henle, 1970; Sumaya *et al*, 1975; Dinand and Arya, 2006). EBV infection is an even more well-established risk factor for Burkitt's lymphoma. There is almost 100% correspondence of EBV presence in endemic Burkitt's lymphoma; sporadic cases are uncommon and the association with EBV is much lower (Kutok and Wang, 2006). International studies indicate, however, that in most developing countries outside Africa, 50–90% of Burkitt's lymphoma cases are EBV positive (Magrath, 1997) leading to speculation that early EBV infection associated with lower SES status and other environmental factors may be relevant in these areas (Klumb *et al*, 2003). This pattern would support our finding of two- to three-fold increased ORs for Burkitt's lymphoma in the lowest category of parental educational attainment. The apparent protective (but non-significant) effect found for Burkitt's lymphoma when comparing children with and without paternal data is puzzling, and simply may be a result of chance and/or small number of cases.

There is little variation in the incidence of Wilm's tumour between developed and developing countries (Bunin, 2004), although an ecological study found evidence of increased Wilm's tumour incidence in more-affluent areas (McNally *et al*, 2003). These results are in contrast to ours; the methodologies used were also quite different and do not allow for direct comparisons. The aetiology of childhood brain tumours remains unclear. The International Agency for Research on Cancer has assembled the largest case–control population for the study of childhood brain tumours to date. With the exception of farm-related exposures and some paternal occupations, little evidence of an association with any environmental factor has been reported (McCredie *et al*, 1999; Filippini *et al*, 2002; Efird *et al*, 2003; Cordier *et al*, 2004; Mueller *et al*, 2004; Cardy *et al*, 2006). Current hypotheses of astrocytoma aetiology do not posit a protective effect of lower SES (Baldwin and Preston-Martin, 2004) and therefore, our finding of lower incidence among children born to mothers with lower educational attainment should be interpreted with reasonable caution.

One study found that parental occupational exposures may increase the risk of hepatoblastoma in offspring (Buckley *et al*, 1989), but the relevant exposures (e.g. metals, petroleum products, paints/pigments) would more likely be encountered in lower education level/manual labour jobs. The association of parental smoking with hepatoblastoma (Pang *et al*, 2003; Sorahan and Lancashire, 2004) may partly reflect uncontrolled confounding from low birth weight (Spector and Ross, 2003), with which there is convincing evidence of a strong inverse association (Spector *et al*, 2008). However, as smoking rates are generally higher among lower SES groups (Orleans, 2003), confounding by smoking would not explain the protective effect suggested for maternal education seen in our data. Our finding of an increased rhabdomyosarcoma OR for children with no paternal data on the birth certificate (i.e. potentially lower SES) mirrors the inverse association of rhabdomyosarcoma with increasing SES indicators found in a small case–control study (Grufferman *et al*, 1982). However, there was no such pattern when specific levels of parental education were evaluated.

Our finding of no pattern of association with childhood leukaemia is of note, given that it has probably been most thoroughly studied for SES associations. Results have been heterogeneous and have varied by measures of SES used, calendar time of study, geographic location and study design (Poole *et al*, 2006; Adam *et al*, 2008) there is no clear evidence of an association between SES and childhood leukaemia.

Our findings suggest that deprivation patterns, that are generally seen when comparing nations of differing economic levels (e.g. Hodgkin's and Burkitt's lymphoma), may be reproduced within the social strata of one nation. Our results should be viewed as exploratory because of the broad nature of the SES assessment, but they do suggest that studies of childhood cancers might benefit from a more thorough evaluation of the child's SES.

## Figures and Tables

**Table 1:** Selected characteristics of cases and controls

**Characteristics**	**Category**	**Cases**	**%**	**Controls**	**%**
Parental characteristics					
Maternal race/ethnicity	Non-Hispanic white	7292	67.6	22172	70.2
	Non-Hispanic black	720	6.7	2078	6.6
	Non-Hispanic Asian	443	4.1	1545	4.9
	Non-Hispanic other	59	0.6	384	1.2
	Hispanic	2267	21.0	5414	17.1
	Missing	1884		7879	
					
Paternal race/ethnicity	Non-Hispanic white	6806	69.0	20137	70.8
	Non-Hispanic black	549	5.6	1664	5.9
	Non-Hispanic Asian	383	3.9	1308	4.6
	Non-Hispanic other	58	0.6	262	0.9
	Hispanic	2067	21.0	5093	17.9
	Missing	2802		11008	
					
Maternal age	<20 years	1240	9.8	4119	10.4
	20–24 years	2972	23.5	9839	24.9
	25–29 years	4049	32.0	12473	31.6
	30–34 years	2957	23.4	8883	22.5
	35+ years	1443	11.4	4148	10.5
	Missing	4		10	
					
Paternal age	<20 years	348	3.1	1104	3.2
	20–24 years	1792	15.9	5964	17.1
	25–29 years	3380	30.0	10453	29.9
	30–34 years	3209	28.5	9785	28.0
	35+ years	2551	22.6	7621	21.8
	Missing	1385		4545	
					
Level of maternal education	<12 years	2482	20.2	7246	19.2
	12 years	4346	35.4	13586	36.1
	13–16 years	4381	35.7	13815	36.7
	17 years or more	1053	8.6	3017	8.0
	Missing	403		1808	
					
Level of paternal education	<12 years	1868	16.8	5013	15.0
	12 years	3937	35.4	12006	35.9
	13–16 years	3997	35.9	12402	37.1
	17 years or more	1330	12.0	4005	12.0
	Missing	1533		6046	
					
Child's characteristics					
Age at diagnosis (cases)	28 days–4 years	8723	68.9		
	5–9 years	2263	17.9		
	10–14 years	1679	13.3		
	Mean age in years (s.d.)	4.5 (3.9)			
					
Sex	Male	7002	55.3	20860	52.9
	Female	5661	44.7	18603	47.1
	Missing	2		9	
					
Plurality	Singleton	12401	97.9	38533	97.6
	Multiple	260	2.1	928	2.4
	Missing	4		11	
					
Birth order	First	5096	41.1	15564	40.7
	Second	4079	32.9	12424	32.5
	Third	1992	16.0	6236	16.3
	Fourth	781	6.3	2400	6.3
	Fifth or higher	465	3.7	1643	4.3
	Missing	252		1205	
	Mean (s.d.)	2.0 (1.2)		2.1 (1.3)	

**Table 2:** Correlations between education measures, overall and by racial/ethnic group

**Measure**	**1**	**2**	**3**
*All*			
1. Maternal education (continuous)	1.00		
2. Paternal education (continuous)	0.71	1.00	
3. Household education (continuous)	0.89	0.88	1.00
			
*Non-Hispanic white* a			
1. Maternal education (continuous)	1.00		
2. Paternal education (continuous)	0.61	1.00	
3. Household education (continuous)	0.85	0.84	1.00
			
*Non-Hispanic black* a			
1. Maternal education (continuous)	1.00		
2. Paternal education (continuous)	0.45	1.00	
3. Household education (continuous)	0.87	0.70	1.00
			
*Hispanic* a			
1. Maternal education (continuous)	1.00		
2. Paternal education (continuous)	0.65	1.00	
3. Household education (continuous)	0.88	0.84	1.00

a Maternal race/ethnicity.

**Table 3:** ORs for educational status and childhood cancer, by ICCC3 major category (adjusted for maternal age, maternal race/ethnicity, parity, state, birth year, gestational age and birth weight)

	**OR (95% CI)**			
**Education level (in years)**	**All cancers**	**Leukemia**	**Lymphoma**	**CNS**
*Maternal*				
<12	1.01 (0.91, 1.13)	1.04 (0.88, 1.24)	1.38 (0.96, 1.99)	0.86 (0.70, 1.05)
12	1.00 (0.91, 1.10)	1.08 (0.93, 1.25)	1.08 (0.79, 1.49)	0.83 (0.70, 0.98)
13–16	0.97 (0.89, 1.06)	1.06 (0.92, 1.22)	1.11 (0.82, 1.52)	0.81 (0.69, 0.96)
>17	*Reference*	*Reference*	*Reference*	*Reference*
				
*Paternal*				
<12	1.12 (1.00, 1.24)	1.09 (0.93, 1.28)	1.21(0.85, 1.73)	1.07 (0.88, 1.31)
12	1.07 (0.98, 1.17)	1.07 (0.94, 1.22)	1.18 (0.88, 1.58)	0.92 (0.78, 1.09)
13–16	1.05 (0.96, 1.14)	1.03 (0.91, 1.17)	1.19 (0.90, 1.58)	0.99 (0.85, 1.16)
>17	*Reference*	*Reference*	*Reference*	*Reference*
				
*Household*				
<12	1.05 (0.95, 1.16)	1.04 (0.90, 1.22)	1.26 (0.90, 1.76)	0.95 (0.78, 1.16)
12	1.03 (0.95, 1.12)	1.02 (0.90, 1.15)	1.00 (0.77, 1.31)	0.92 (0.79, 1.08)
13–16	1.00 (0.93, 1.08)	1.01 (0.90, 1.13)	1.15 (0.90, 1.46)	0.94 (0.82, 1.07)
>17	*Reference*	*Reference*	*Reference*	*Reference*
				
	**Sympathetic nervous system**	**Retinoblastoma**	**Renal tumours**	**Hepatic tumours**
*Maternal*				
<12	0.94 (0.70, 1.26)	1.27 (0.83, 1.94)	1.26 (0.90, 1.78)	0.85 (0.48, 1.49)
12	1.03 (0.81, 1.32)	1.17 (0.79, 1.72)	1.29 (0.95, 1.73)	0.70 (0.43, 1.14)
13–16	1.04 (0.83, 1.31)	1.13 (0.78, 1.65)	1.22 (0.91, 1.62)	0.67 (0.42, 1.06)
>17	*Reference*	*Reference*	*Reference*	*Reference*
				
*Paternal*				
<12	1.07 (0.81, 1.42)	1.48 (0.98, 2.24)	1.38 (1.00, 1.91)	1.01 (0.56, 1.81)
12	1.10 (0.88, 1.38)	1.45 (1.01, 2.10)	1.32 (1.01, 1.73)	1.00 (0.61, 1.64)
13–16	1.08 (0.87, 1.34)	1.31 (0.91, 1.87)	1.13 (0.87, 1.47)	0.93 (0.58, 1.49)
>17	*Reference*	*Reference*	*Reference*	*Reference*
				
*Household*				
<12	1.00 (0.77, 1.32)	1.20 (0.82, 1.75)	1.25 (0.91, 1.71)	0.94 (0.54, 1.62)
12	1.07 (0.87, 1.32)	1.29 (0.94, 1.78)	1.32 (1.03, 1.70)	0.95 (0.61, 1.49)
13–16	1.02 (0.84, 1.23)	1.17 (0.86, 1.58)	1.21 (0.96, 1.52)	0.80 (0.53, 1.21)
>17	*Reference*	*Reference*	*Reference*	*Reference*
				
	**Malignant bone**	**Soft-tissue sarcomas**	**Germ cell**	**Other carcinomas**
*Maternal*				
<12	0.88 (0.49, 1.61)	1.03 (0.71, 1.49)	1.08 (0.66, 1.79)	1.56 (0.72, 3.36)
12	0.71 (0.44, 1.14)	0.96 (0.70, 1.32)	1.01 (0.65, 1.57)	1.23 (0.66, 2.28)
13–16	0.69 (0.44, 1.09)	0.92 (0.68, 1.24)	0.83 (0.54, 1.28)	0.79 (0.42, 1.46)
>17	*Reference*	*Reference*	*Reference*	*Reference*
				
*Paternal*				
<12	1.40 (0.77, 2.55)	1.09 (0.75, 1.59)	0.97 (0.60, 1.57)	0.75 (0.31, 1.77)
12	0.94 (0.58, 1.52)	1.07 (0.79, 1.45)	1.02 (0.68, 1.53)	1.37 (0.79, 2.37)
13–16	1.15 (0.74, 1.80)	1.14 (0.85, 1.51)	0.96 (0.65, 1.42)	0.75 (0.42, 1.31)
>17	*Reference*	*Reference*	*Reference*	*Reference*
				
*Household*				
<12	1.23 (0.68, 2.24)	1.16 (0.82, 1.66)	1.00 (0.63, 1.59)	1.51 (0.70, 3.27)
12	0.89 (0.57, 1.37)	1.05 (0.80, 1.39)	1.03 (0.71, 1.50)	1.50 (0.88, 2.55)
13–16	0.94 (0.64, 1.38)	1.07 (0.84, 1.38)	0.84 (0.59, 1.19)	0.86 (0.51, 1.43)
>17	*Reference*	*Reference*	*Reference*	*Reference*

Abbreviations: CI=confidence interval; CNS=central nervous system; ICCC3=International Classification of Childhood Cancer third edition; OR=odds ratio.

**Table 4:** Risk of childhood cancer associated with missing paternal data as SES indicator, by cancer type^a^

**Cancer type**	**No. of cases with no paternal data**	**OR**	**95% CI**	
*Total cancers*	920	0.95	0.86	1.04
*Leukemia*	262	0.86	0.74	1.01
ALL	209	0.86	0.72	1.03
AML	38	0.82	0.56	1.20
				
*Lymphoma*	71	0.80	0.59	1.09
Hodgkin's lymphoma	22	1.06	0.60	1.87
NHL	27	0.86	0.55	1.37
Burkitt's	9	0.47	0.20	1.12
				
*CNS*	183	0.86	0.72	1.04
Ependymoma	18	0.64	0.36	1.13
Astrocytoma	86	1.00	0.76	1.31
Intracranial embryonal	39	0.81	0.55	1.17
Other gliomas	26	0.88	0.54	1.44
				
*Sympathetic nervous system*	94	1.10	0.87	1.40
Neuroblastoma	93	1.10	0.86	1.40
				
Retinoblastoma	49	1.01	0.71	1.42
				
*Renal tumours*	81	0.99	0.75	1.29
Wilm's tumor	79	0.97	0.74	1.28
				
*Hepatic tumours*	30	1.23	0.77	1.97
Hepatoblastoma	24	1.14	0.68	1.90
				
*Bone tumours*	25	1.10	0.66	1.85
Osteosarcoma	15	0.90	0.43	1.89
Ewing's sarcoma	6	0.87	0.34	2.22
				
*Soft-tissue sarcomas*	73	1.40	1.05	1.87
Rhabdomyosarcoma	45	1.59	1.11	2.29
				
*Germ-cell tumours*	30	0.91	0.58	1.43
Extracranial germ-cell tumours	8	0.95	0.42	2.18
Gonadal germ-cell tumours	15	1.06	0.58	1.91
*Other epithelial*	15	1.04	0.54	2.01

Abbreviations: ALL=acute lymphoblastic leukemia; AML=acute myeloid leukemia; CI=confidence interval; CNS=central nervous system; NHL=non-Hodgkin's lymphoma; OR=odds ratio; SES=socioeconomic status.

a Observations with some paternal data as referent; adjusted for maternal age group, maternal race/ethnicity, sex, state, birth year, birth order, birth weight and gestational age.

## References

[bib1] Adam M, Rebholz CE, Egger M, Zwahlen M, Kuehni CE (2008) Childhood leukaemia and socioeconomic status: what is the evidence? Radiat Prot Dosimetry 132(2): 246–254 18927134 10.1093/rpd/ncn261

[bib2] Baldwin RT, Preston-Martin S (2004) Epidemiology of brain tumors in childhood--a review. Toxicol Appl Pharmacol 199(2): 118–131 15313584 10.1016/j.taap.2003.12.029

[bib3] Braveman PA, Cubbin C, Egerter S, Chideya S, Marchi KS, Metzler M, Posner S (2005) Socioeconomic status in health research: one size does not fit all. JAMA 294(22): 2879–2888 16352796 10.1001/jama.294.22.2879

[bib4] Buckley JD, Sather H, Ruccione K, Rogers PC, Haas JE, Henderson BE, Hammond GD (1989) A case-control study of risk factors for hepatoblastoma. A report from the Childrens Cancer Study Group. Cancer 64(5): 1169–1176 2547509 10.1002/1097-0142(19890901)64:5<1169::aid-cncr2820640534>3.0.co;2-i

[bib5] Bunin GR (2004) Nongenetic causes of childhood cancers: evidence from international variation, time trends, and risk factor studies. Toxicol Appl Pharmacol 199(2): 91–103 15313582 10.1016/j.taap.2003.12.028

[bib6] Cardy AH, Little J, McKean-Cowdin R, Lijinsky W, Choi NW, Cordier S, Filippini G, Holly EA, Lubin F, McCredie M, Mueller BA, Peris-Bonet R, Arslan A, Preston-Martin S (2006) Maternal medication use and the risk of brain tumors in the offspring: the SEARCH international case-control study. Int J Cancer 118(5): 1302–1308 16161045 10.1002/ijc.21482

[bib7] Carozza SE, Li B, Wang Q, Horel S, Cooper S (2009) Agricultural pesticides and risk of childhood cancers. Int J Hyg Environ Health 212(2): 186–195 18675586 10.1016/j.ijheh.2008.06.002 PMC2659586

[bib8] Cordier S, Monfort C, Filippini G, Preston-Martin S, Lubin F, Mueller BA, Holly EA, Peris-Bonet R, McCredie M, Choi W, Little J, Arslan A (2004) Parental exposure to polycyclic aromatic hydrocarbons and the risk of childhood brain tumors: the SEARCH International Childhood Brain Tumor Study. Am J Epidemiol 159(12): 1109–1116 15191928 10.1093/aje/kwh154

[bib9] Correa P, O’Conor GT (1971) Epidemiologic patterns of Hodgkin's disease. Int J Cancer 8(2): 192–201 5133848 10.1002/ijc.2910080203

[bib10] Daly MC, Duncan GJ, McDonough P, Williams DR (2002) Optimal indicators of socioeconomic status for health research. Am J Public Health 92(7): 1151–1157 12084700 10.2105/ajph.92.7.1151 PMC1447206

[bib11] Dinand V, Arya LS (2006) Epidemiology of childhood Hodgkin's disease: is it different in developing countries? Indian Pediatr 43(2): 141–147 16528110

[bib12] Efird JT, Holly EA, Preston-Martin S, Mueller BA, Lubin F, Filippini G, Peris-Bonet R, McCredie M, Cordier S, Arslan A, Bracci PM (2003) Farm-related exposures and childhood brain tumours in seven countries: results from the SEARCH International Brain Tumour Study. Paediatr Perinat Epidemiol 17(2): 201–211 12675788 10.1046/j.1365-3016.2003.00484.x

[bib13] Filippini G, Maisonneuve P, McCredie M, Peris-Bonet R, Modan B, Preston-Martin S, Mueller BA, Holly EA, Cordier S, Choi NW, Little J, Arslan A, Boyle P (2002) Relation of childhood brain tumors to exposure of parents and children to tobacco smoke: the SEARCH international case-control study. Surveillance of environmental aspects related to cancer in humans. Int J Cancer 100(2): 206–213 12115571 10.1002/ijc.10465

[bib14] Galobardes B, Lynch J, Smith GD (2007) Measuring socioeconomic position in health research. Br Med Bull 81-82: 21–37 17284541 10.1093/bmb/ldm001

[bib15] Galobardes B, Shaw M, Lawlor DA, Lynch JW, Davey Smith G (2006) Indicators of socioeconomic position (part 1). J Epidemiol Community Health 60(1): 7–12 10.1136/jech.2004.023531 PMC2465546 16361448

[bib16] Grufferman S, Wang HH, DeLong ER, Kimm SY, Delzell ES, Falletta JM (1982) Environmental factors in the etiology of rhabdomyosarcoma in childhood. J Natl Cancer Inst 68(1): 107–113 6948120

[bib17] Gutensohn NM, Shapiro DS (1982) Social class risk factors among children with Hodgkin's disease. Int J Cancer 30(4): 433–435 7141739 10.1002/ijc.2910300409

[bib18] Hauser RM (1994) Measuring socioeconomic status in studies of child development. Child Dev 65(6): 1541–1545 7859541 10.1111/j.1467-8624.1994.tb00834.x

[bib19] Henle G, Henle W (1970) Observations on childhood infections with the Epstein-Barr virus. J Infect Dis 121(3): 303–310 4313278 10.1093/infdis/121.3.303

[bib20] Jaro MA (1995) Probabilistic linkage of large public health data files. Stat Med 14(5–7): 491–498 7792443 10.1002/sim.4780140510

[bib21] Klumb CE, de Resende LM, Stefanoff CG, Vicuna CH, Renault IZ, Maia RC (2003) Burkitt-like lymphoma in an infant: a case report. Rev Hosp Clin Fac Med Sao Paulo 58(1): 33–36 12754588 10.1590/s0041-87812003000100007

[bib22] Krieger N, Williams DR, Moss NE (1997) Measuring social class in US public health research: concepts, methodologies, and guidelines. Annu Rev Public Health 18: 341–378 9143723 10.1146/annurev.publhealth.18.1.341

[bib23] Kutok JL, Wang F (2006) Spectrum of Epstein-Barr virus-associated diseases. Annu Rev Pathol 1: 375–404 18039120 10.1146/annurev.pathol.1.110304.100209

[bib24] Magrath IT (1997) Non-Hodgkin's lymphomas: epidemiology and treatment. Ann NY Acad Sci 824: 91–106 9382459 10.1111/j.1749-6632.1997.tb46212.x

[bib25] McCredie M, Little J, Cotton S, Mueller B, Peris-Bonet R, Choi NW, Cordier S, Filippini G, Holly EA, Modan B, Arslan A, Preston-Martin S (1999) SEARCH international case-control study of childhood brain tumours: role of index pregnancy and birth, and mother's reproductive history. Paediatr Perinat Epidemiol 13(3): 325–341 10440052 10.1046/j.1365-3016.1999.00195.x

[bib26] McLaughlin CC, Baptiste MS, Schymura MJ, Nasca PC, Zdeb MS (2006) Maternal and infant birth characteristics and hepatoblastoma. Am J Epidemiol 163(9): 818–828 16510543 10.1093/aje/kwj104

[bib27] McNally RJ, Alston RD, Cairns DP, Eden OB, Kelsey AM, Birch JM (2003) Geographical and ecological analyses of childhood Wilms’ tumours and soft-tissue sarcomas in North West England. Eur J Cancer 39(11): 1586–1593 12855266 10.1016/s0959-8049(03)00357-5

[bib28] Mueller BA, Nielsen SS, Preston-Martin S, Holly EA, Cordier S, Filippini G, Peris-Bonet R, Choi NW (2004) Household water source and the risk of childhood brain tumours: results of the SEARCH International Brain Tumor Study. Int J Epidemiol 33(6): 1209–1216 15567873 10.1093/ije/dyh215

[bib29] Northam S, Knapp TR (2006) The reliability and validity of birth certificates. J Obstet Gynecol Neonatal Nurs 35(1): 3–12 10.1111/j.1552-6909.2006.00016.x 16466348

[bib30] Orleans CT (2003) Those who continue to smoke: is achieving abstinence harder and do we need to change our interventions?. In Smoking and Tobacco Control Monograph No 15 NC Institute (ed), US Department of Health and Human Services, Public Health Service, National Institutes of Health, National Cancer Institute: 1–9

[bib31] Pang D, McNally R, Birch JM (2003) Parental smoking and childhood cancer: results from the United Kingdom Childhood Cancer Study. Br J Cancer 88(3): 373–381 12569379 10.1038/sj.bjc.6600774 PMC2747546

[bib32] Podvin D, Kuehn CM, Mueller BA, Williams M (2006) Maternal and birth characteristics in relation to childhood leukaemia. Paediatr Perinat Epidemiol 20(4): 312–322 16879503 10.1111/j.1365-3016.2006.00731.x

[bib33] Poole C, Greenland S, Luetters C, Kelsey JL, Mezei G (2006) Socioeconomic status and childhood leukaemia: a review. Int J Epidemiol 35(2): 370–384 16308412 10.1093/ije/dyi248

[bib34] Puumala SE, Carozza SE, Chow EJ, Fox EE, Horel S, Johnson KJ, McLaughlin C, Mueller BA, Reynolds P, Von Behren J, Spector LG (2009) Childhood cancer among twins and higher order multiples. Cancer Epidemiol Biomarkers Prev 18(1): 162–168 19124494 10.1158/1055-9965.EPI-08-0660 PMC2705199

[bib35] Puumala SE, Soler JT, Johnson KJ, Spector LG (2008) Birth characteristics and Wilms tumor in Minnesota. Int J Cancer 122(6): 1368–1373 18033684 10.1002/ijc.23275

[bib36] Querec LJ (1980) Comparability of reporting between the birth certificate and the National Natality Survey. Vital Health Stat 2(83): 1–44 7395112

[bib37] Reynolds P, Von Behren J, Elkin EP (2002) Birth characteristics and leukemia in young children. Am J Epidemiol 155(7): 603–613 11914187 10.1093/aje/155.7.603

[bib38] Seeman TE, Crimmins E (2001) Social environment effects on health and aging: integrating epidemiologic and demographic approaches and perspectives. Ann N Y Acad Sci 954: 88–117 11797869 10.1111/j.1749-6632.2001.tb02749.x

[bib39] Sorahan T, Lancashire RJ (2004) Parental cigarette smoking and childhood risks of hepatoblastoma: OSCC data. Br J Cancer 90(5): 1016–1018 14997199 10.1038/sj.bjc.6601651 PMC2409629

[bib40] Spector LG, Johnson KJ, Soler JT, Puumala SE (2008) Perinatal risk factors for hepatoblastoma. Br J Cancer 98(9): 1570–1573 18392049 10.1038/sj.bjc.6604335 PMC2391098

[bib41] Spector LG, Ross JA (2003) Smoking and hepatoblastoma: confounding by birth weight? Br J Cancer 89(3): 602; author reply 603 12888836 10.1038/sj.bjc.6601143 PMC2394388

[bib42] Steliarova-Foucher E, Stiller C, Lacour B, Kaatsch P (2005) International Classification of Childhood Cancer, third edition. Cancer 103(7): 1457–1467 15712273 10.1002/cncr.20910

[bib43] Stiller CA (1998) What causes Hodgkin's disease in children? Eur J Cancer 34(4): 523–528 9713303 10.1016/s0959-8049(97)10096-x

[bib44] Sumaya CV, Henle W, Henle G, Smith MH, LeBlanc D (1975) Seroepidemiologic study of Epstein-Barr virus infections in a rural community. J Infect Dis 131(4): 403–408 163869 10.1093/infdis/131.4.403

[bib45] Tan H, Wen SW, Walker M, Demissie K (2004) Missing paternal demographics: a novel indicator for identifying high risk population of adverse pregnancy outcomes. BMC Pregnancy Childbirth 4(1): 21 15541183 10.1186/1471-2393-4-21 PMC535341

[bib46] Zill N (1996) Parental schooling & children's health. Public Health Rep 111(1): 34–43 8610189 PMC1381739

