# Supplementary material for: Parental educational attainment as an indicator of socioeconomic status and risk of childhood cancers
Source: Br J Cancer. 2010 Jun 8;103(1):136–42. doi: 10.1038/sj.bjc.6605732 (PMC2905284; doi:10.1038/sj.bjc.6605732)
Supplement: Supplementary Table [file 6605732x1.doc]

| Table 3 (supplemental). Odds ratios for educational status and childhood cancer sites with >200 cases (adjusted for maternal age, maternal race/ethnicity, parity, state, birth year, gestational age, and birth weight). | | | | | | | | | |
| --- | --- | --- | --- | --- | --- | --- | --- | --- | --- |
|  | | OR (95% CI) | | | | | | | |
| Education level  (in years) | | All Cancers | Leukemia | Lymphoid leukemia | AML | Lymphoma | Hodgkin lymphoma | NHL | Burkitt lymphoma |
| Maternal | <12 | 1.01 (0.91,1.13) | 1.04 (0.88,1.24) | 1.05 (0.87,1.26) | 1.03 (0.67,1.57) | 1.38 (0.96,1.99) | 2.03 (0.87,4.77) | 1.18 (0.69,2.03) | 3.32 (1.26,8.71) |
|  | 12 | 1.00 (0.91,1.10) | 1.08 (0.93,1.25) | 1.04 (0.89,1.22) | 1.30 (0.90,1.88) | 1.08 (0.79,1.49) | 1.20 (0.55,2.63) | 1.08 (0.68,1.69) | 1.94 (0.80,4.72) |
|  | 13-16 | 0.97 (0.89,1.06) | 1.06 (0.92 1.22) | 1.05 (0.90, 1.22) | 1.14 (0.80,1.63) | 1.11 (0.82,1.52) | 1.67 (0.79,3.55) | 1.04 (0.67,1.61) | 1.71 (0.72,4.09) |
|  | >17 | Reference | Reference | Reference | Reference | Reference | Reference | Reference | Reference |
| Paternal | <12 | 1.12 (1.00, 1.24) | 1.09 (0.93,1.28) | 1.00 (0.84,1.19) | 1.26 (0.85,1.87) | 1.21( 0.85,1.73) | 1.53 (0.74,3.20) | 1.37 (0.80,2.34) | 2.00 (0.80,4.95) |
|  | 12 | 1.07 (0.98, 1.17) | 1.07 (0.94,1.22) | 0.99 (0.86,1.15) | 1.38 (0.99,1.91) | 1.18 (0.88,1.58) | 1.08 (0.58,2.03) | 1.28 (0.82,1.99) | 2.06 (0.94,4.51) |
|  | 13-16 | 1.05 (0.96, 1.14) | 1.03 (0.91,1.17) | 0.99 (0.87,1.14) | 1.05 (0.76,1.46) | 1.19 (0.90,1.58) | 1.25 (0.69,2.29) | 1.36 (0.89,2.08) | 1.81 (0.84,3.90) |
|  | >17 | Reference | Reference | Reference | Reference | Reference | Reference | Reference | Reference |
| Household | <12 | 1.05 (0.95, 1.16) | 1.04 (0.90,1.22) | 1.01 (0.85,1.19) | 1.08 (0.74,1.58) | 1.26 (0.90,1.76) | 2.17 (1.07,4.38) | 1.22 (0.73,2.03) | 1.38 (0.58,3.29) |
|  | 12 | 1.03 (0.95, 1.12) | 1.02 (0.90,1.15) | 0.97 (0.85,1.11) | 1.25 (0.92,1.68) | 1.00 (0.77,1.31) | 1.02 (0.56,1.87) | 1.02 (0.68,1.52) | 1.67 (0.84,3.33) |
|  | 13-16 | 1.00 (0.93, 1.08) | 1.01 (0.90,1.13) | 0.98 (0.87,1.11) | 1.05 (0.79,1.38) | 1.15 (0.90,1.46) | 1.42 (0.82,2.45) | 1.18 (0.83,1.69) | 1.74 (0.92,3.30) |
|  | >17 | Reference | Reference | Reference | Reference | Reference | Reference | Reference | Reference |
|  |  |  |  |  |  |  |  |  |  |
|  |  | CNS | Ependymoma | Astrocytoma | Intracranial embryonal | Other gliomas | Sympathetic nervous system | Neuroblastoma | Retinoblastoma |
| Maternal | <12 | 0.86 (0.70,1.05) | 1.08 (0.60,1.96) | 0.70 (0.51,0.95) | 0.98 (0.66,1.44) | 1.00 (0.57,1.75) | 0.94 (0.70,1.26) | 0.95 (0.71,1.27) | 1.27 (0.83,1.94) |
|  | 12 | 0.83 (0.70,0.98) | 0.99 (0.59,1.66) | 0.88 (0.69,1.13) | 0.74 (0.52,1.04) | 0.90 (0.56,1.43) | 1.03 (0.81,1.32) | 1.05 (0.82,1.34) | 1.17 (0.79,1.72) |
|  | 13-16 | 0.81 (0.69, 0.96) | 0.97 (0.59,1.59) | 0.81 (0.64,1.03) | 0.85 (0.62,1.18) | 0.83 (0.53,1.30) | 1.04 (0.83,1.31) | 1.06 (0.84,1.33) | 1.13 (0.78,1.65) |
|  | >17 | Reference | Reference | Reference | Reference | Reference | Reference | Reference | Reference |
| Paternal | <12 | 1.07 (0.88,1.31) | 0.98 (0.55,1.73) | 1.08 (0.80,1.48) | 1.05 (0.72,1.53) | 1.44 (0.85,2.43) | 1.07 (0.81,1.42) | 1.09 (0.82,1.44) | 1.48 (0.98,2.24) |
|  | 12 | 0.92 (0.78,1.09) | 0.95 (0.59,1.50) | 1.02 (0.80,1.30) | 0.83 (0.60,1.15) | 0.92 (0.59,1.43) | 1.10 (0.88,1.38) | 1.10 (0.88,1.38) | 1.45 (1.01,2.10) |
|  | 13-16 | 0.99 (0.85,1.16) | 0.97 (0.63,1.51) | 1.09 (0.87,1.37) | 0.95 (0.70,1.30) | 0.88 (0.58,1.35) | 1.08 (0.87,1.34) | 1.09 (0.88,1.36) | 1.31 (0.91,1.87) |
|  | >17 | Reference | Reference | Reference | Reference | Reference | Reference | Reference | Reference |
| Household | <12 | 0.95 (0.78,1.16) | 1.04 (0.59,1.82) | 0.82 (0.60,1.11) | 0.99 (0.68,1.43) | 1.46 (0.87,2.45) | 1.00 (0.77,1.32) | 1.01 (0.77,1.32) | 1.20 (0.82,1.75) |
|  | 12 | 0.92 (0.79,1.08) | 1.05 (0.67,1.62) | 0.97 (0.78,1.21) | 0.87 (0.64,1.18) | 0.99 (0.65,1.50) | 1.07 (0.87,1.32) | 1.07 (0.86,1.32) | 1.29 (0.94,1.78) |
|  | 13-16 | 0.94 (0.82,1.07) | 1.00 (0.68,1.49) | 0.95 (0.78,1.16) | 0.99 (0.75,1.29) | 0.95 (0.65,1.38) | 1.02 (0.84,1.23) | 1.03 (0.85,1.24) | 1.17 (0.86,1.58) |
|  | >17 | Reference | Reference | Reference | Reference | Reference | Reference | Reference | Reference |
|  |  |  |  |  |  |  |  |  |  |
|  |  | Renal tumors | Wilms tumor | Hepatic tumors | Hepatoblastoma | Malignant bone | Osteosarcoma | Ewing sarcoma | Soft-tissue sarcomas |
| Maternal | <12 | 1.26 (0.90,1.78) | 1.23 (0.87,1.74) | 0.85 (0.48,1.49) | 0.80 (0.45,1.43) | 0.88 (0.49,1.61) | 0.80 (0.30,2.16) | 1.00 (0.40,2.46) | 1.03 (0.71,1.49) |
|  | 12 | 1.29 (0.95,1.73) | 1.26 (0.93,1.69) | 0.70 (0.43,1.14) | 0.59 (0.36,0.98) | 0.71 (0.44,1.14) | 0.71 (0.32,1.58) | 0.92 (0.47,1.79) | 0.96 (0.70,1.32) |
|  | 13-16 | 1.22 (0.91,1.62) | 1.19 (0.90,1.59) | 0.67 (0.42,1.06) | 0.59 (0.37,0.94) | 0.69 (0.44,1.09) | 0.99 (0.46,2.09) | 0.60 (0.30,1.17) | 0.92 (0.68,1.24) |
|  | >17 | Reference | Reference | Reference | Reference | Reference | Reference | Reference | Reference |
| Paternal | <12 | 1.38 (1.00,1.91) | 1.45 (1.04,2.02) | 1.01 (0.56,1.81) | 1.09 (0.60,2.01) | 1.40 (0.77,2.55) | 1.59 (0.64,3.91) | 1.86 (0.73,4.72) | 1.09 (0.75,1.59) |
|  | 12 | 1.32 (1.01,1.73) | 1.39 (1.06,1.83) | 1.00 (0.61,1.64) | 1.02 (0.61,1.71) | 0.94 (0.58,1.52) | 0.90 (0.43,1.90) | 1.16 (0.54,2.48) | 1.07 (0.79,1.45) |
|  | 13-16 | 1.13 (0.87,1.47) | 1.18 (0.90,1.54) | 0.93 (0.58,1.49) | 0.88 (0.54,1.45) | 1.15 (0.74,1.80) | 1.01 (0.50,2.04) | 1.57 (0.78,3.17) | 1.14 (0.85,1.51) |
|  | >17 | Reference | Reference | Reference | Reference | Reference | Reference | Reference | Reference |
| Household | <12 | 1.25 (0.91,1.71) | 1.28 (0.93,1.76) | 0.94 (0.54,1.62) | 0.95 (0.54,1.66) | 1.23 (0.68,2.24) | 1.35 (0.56,3.28) | 1.36 (0.50,3.69) | 1.16 (0.82,1.66) |
|  | 12 | 1.32 (1.03,1.70) | 1.36 (1.05,1.75) | 0.95 (0.61,1.49) | 0.89 (0.56, 1.42) | 0.89 (0.57,1.37) | 0.87 (0.44,1.70) | 1.25 (0.64,2.44) | 1.05 (0.80,1.39) |
|  | 13-16 | 1.21 (0.96,1.52) | 1.24 (0.98,1.57) | 0.80 (0.53,1.21) | 0.74 (0.48,1.13) | 0.94 (0.64,1.38) | 0.90 (0.49,1.65) | 1.24 (0.68,2.26) | 1.07 (0.84,1.38) |
|  | >17 | Reference | Reference | Reference | Reference | Reference | Reference | Reference | Reference |
|  |  |  |  |  |  |  |  |  |  |
|  |  | Rhabdomyosarcoma | Germ-cell | Extracranial GCT | Gonadal GCT | Carcinomas |  |  |  |
| Maternal | <12 | 0.83 (0.52,1.32) | 1.08 (0.66,1.79) | 0.70 (0.31,1.61) | 1.99 (0.87,4.56) | 1.56 (0.72,3.36) |  |  |  |
|  | 12 | 0.80 (0.55,1.17) | 1.01 (0.65,1.57) | 0.77 (0.39,1.54) | 1.58 (0.73,3.46) | 1.23 (0.66,2.28) |  |  |  |
|  | 13-16 | 0.81 (0.57,1.16) | 0.83 (0.54,1.28) | 0.51 (0.26,1.00) | 1.62 (0.76,3.45) | 0.79 (0.42,1.46) |  |  |  |
|  | >17 | Reference | Reference | Reference | Reference | Reference |  |  |  |
| Paternal | <12 | 0.98 (0.61,1.57) | 0.97 (0.60,1.57) | 0.87 (0.37,2.01) | 1.21 (0.61,2.40) | 0.75 (0.31,1.77) |  |  |  |
|  | 12 | 0.95 (0.65,1.38) | 1.02 (0.68,1.53) | 0.73 (0.36,1.49) | 1.19 (0.65,2.20) | 1.37 (0.79,2.37) |  |  |  |
|  | 13-16 | 1.00 (0.71,1.42) | 0.96 (0.65,1.42) | 0.89 (0.46,1.71) | 1.06 (0.59,1.94) | 0.75 (0.42,1.31) |  |  |  |
|  | >17 | Reference | Reference | Reference | Reference | Reference |  |  |  |
| Household | <12 | 1.04 (0.67,1.64) | 1.00 (0.63,1.59) | 0.77 (0.35,1.72) | 1.36 (0.71,2.62) | 1.51 (0.70,3.27) |  |  |  |
|  | 12 | 0.89 (0.63,1.27) | 1.03 (0.71,1.50) | 0.77 (0.41,1.45) | 1.22 (0.69,2.17) | 1.50 (0.88,2.55) |  |  |  |
|  | 13-16 | 0.99 (0.73,1.34) | 0.84 (0.59,1.19) | 0.63 (0.35,1.12) | 1.11 (0.65,1.90) | 0.86 (0.51,1.43) |  |  |  |
|  | >17 | Reference | Reference | Reference | Reference | Reference |  |  |  |

Abbreviations: AML=acute myeloid leukemias; NHL=Non-Hodgkin lymphomas; CNS=central nervous system; GCT=germ cell tumors
